# A Child with Severe Malaria Presenting with Acute Surgical Abdomen (Duodenal Perforation)

**DOI:** 10.1155/2016/3092130

**Published:** 2016-10-23

**Authors:** Tika Ram Bhandari, Sudha Shahi, Rajesh Poudel, Nagendra Chaudhary

**Affiliations:** ^1^Department of General Surgery, Universal College of Medical Sciences, Bhairahawa 32900, Nepal; ^2^Department of ENT, National Academy of Medical Sciences, Kathmandu 44600, Nepal; ^3^Department of Pediatrics, Universal College of Medical Sciences, Bhairahawa 32900, Nepal

## Abstract

*Plasmodium falciparum*, the commonest cause of severe malaria in children, is an important cause of mortality in developing nations like Nepal. Duodenal perforation in a case of complicated malaria, although a rare entity, can occur in children. Early diagnosis, proper medical treatment, and early surgical repair can be a lifesaving measure in such cases. Here, we report a case of a 5-year-old male child with* falciparum* malaria complicated by a duodenal perforation that was successively managed with appropriate antimalarial drugs and early surgical repair.

## 1. Introduction

Duodenal perforation in children is a rare surgical emergency often missed or not considered commonly in the differential diagnoses of acute abdomen [1, 2]. Peptic ulcer disease (PUD) in children is reported worldwide with an estimated frequency of 8.1% in Europe and 17.4% in the US, thus remaining relatively rare as compared with adults. Malaria is one of the major public health problems particularly in developing countries. Among the age groups, young children are at highest risk. Severe malaria caused by* P. falciparum* can present with unarousable coma, loss of consciousness, metabolic acidosis, hypoglycemia, increased lactate, jaundice, acute kidney injury, cardiac failure, hyperparasitemia, and pulmonary edema [3]. Abdominal symptoms like vomiting, dyspepsia, abdominal pain, intestinal hemorrhage, and obstruction are well known in acute malaria [4–7]. Rarely perforation can also occur. Reports on intestinal perforations associated with malaria are few.

## 2. Case Presentation

**Figure 1:**
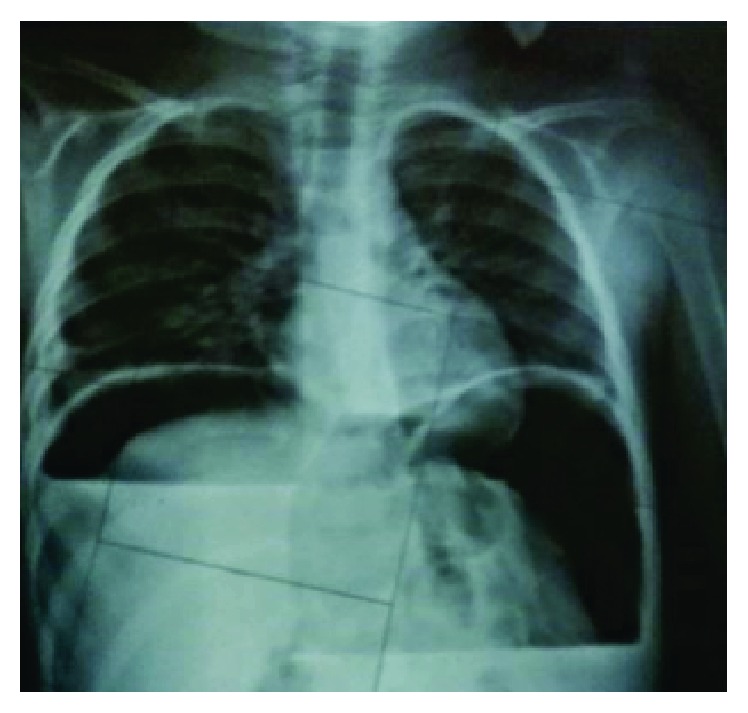
Showing gas under diaphragm.

**Figure 2:**
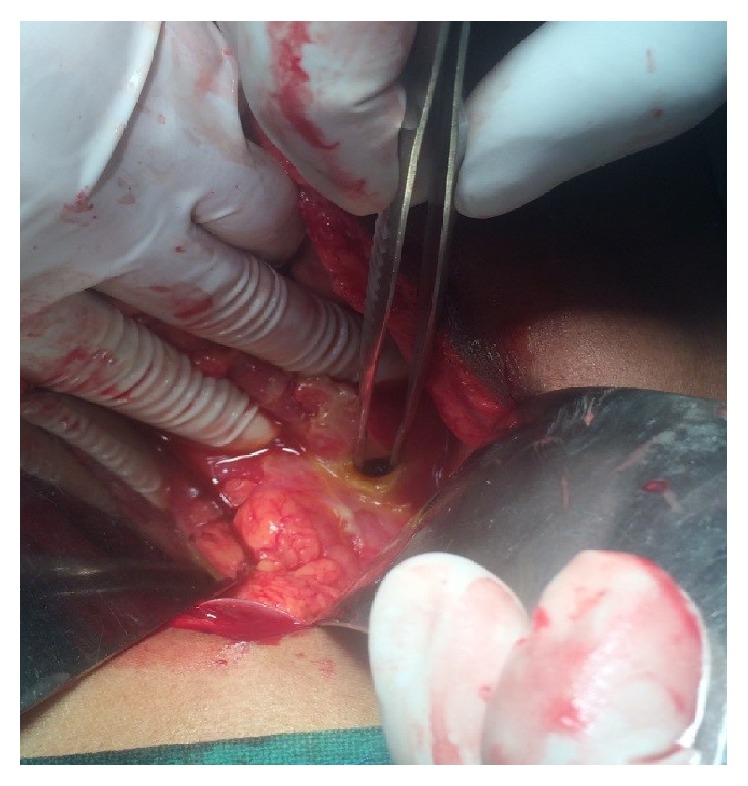
Showing duodenal perforation.

**Figure 3:**
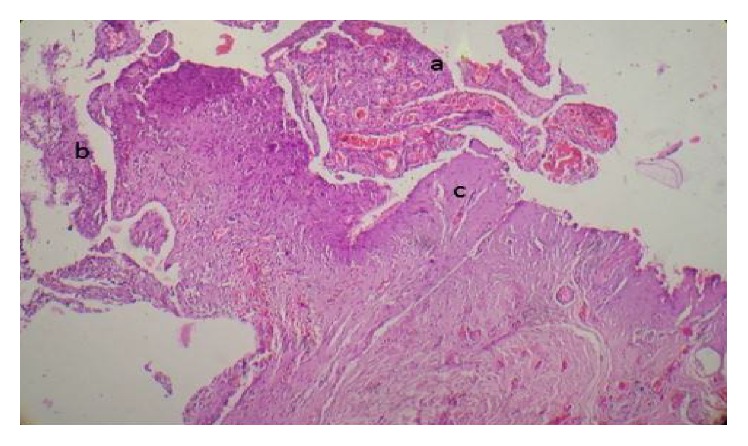
Perforated site ulceration showing granulation tissue (a), necrosis and chronic inflammation with activity (b), and fibrous areas (c).

A 5-year-old male child was admitted with history of high grade (102.6°F), intermittent fever associated with loose motions, and vomiting for 3 days. He developed severe abdomen pain associated with distension 2 days prior to admission. There was no history of any drug intake prior to admission except WHO oral rehydration solution. On admission, he had signs of severe dehydration (lethargy, skin pinch >3 seconds, dry tongue, and conjunctiva). On examination, child had clinical features suggestive of shock (blood pressure <3rd centile, cool extremities, weak and thready pulse, capillary refill time >3 seconds, and tachycardia). Abdomen examination revealed distension with features of peritonitis. Child received 3 fluid boluses (normal saline at 20 mL/kg, each) and inotropes along with intravenous antibiotics. On investigation, hemoglobin was 10 gm/dL, white blood cells were 25000 cells/mm^3^, and platelet count was 90,000 cells/mm^3^. Peripheral smear (both thick and thin) was positive for malarial parasite (*P. falciparum*) with parasite load of 190/*μ*L. Antigen test for the same was positive for* Plasmodium falciparum*. Child was started on intravenous artesunate and clindamycin as per WHO guidelines. Plain X-ray abdomen showed gas under diaphragm (Figure 1). Child was considered for laparotomy and further proceed. During laparotomy, midline incision released a large amount of free gas along with 500 mL of bile stained fluid contaminated with food. The stomach and duodenum were exposed and a perforated duodenal ulcer, 0.5 cm in diameter, was found (Figure 2) and surgically closed with omental patch. The postoperative course was uneventful. Histopathology of duodenal mucosa (Figure 3) was negative for* H. pylori*. Repeat malarial parasite load was nil. He was discharged on day 10 after surgery.

## 3. Discussion

Peptic ulcers which rarely occur in pediatric age groups can be of primary or secondary etiology. Primary ulcers have a male preponderance (male/female 4 : 1), often present at a mean age of 8 years, occur commonly in children having blood group O, and are associated with* Helicobacter pylori* infection [1, 8]. Secondary ulcers are precipitated by physiological stress, severe burns (Curling's ulcer), raised intracranial pressure (Cushing's ulcer) [9], drugs (steroids, nonsteroidal anti-inflammatory drugs), and other severe illnesses (gastroenteritis, shock, sepsis, or cancer) [10–13]. Primary peptic ulcers rarely present with perforation whereas secondary ulcers present commonly with perforation and hemorrhage. The majority of ulcers occur in infants (80%) with the rest occurring in older age groups (20%). The most common causes for duodenal perforation in young children are accidental injury or trauma caused by child abuse.

Duodenal perforation in children has been reported comprising various etiologies [14]. Literature on duodenal perforation in malaria is rare with few case reports published sporadically. Goldman et al. recently reported a healthy twelve-month child with severe malaria who presented with perforated duodenal ulcer [8]. Dewanda and Midya from India too reported a similar case in a 4-year-old male child who was successively treated with omental patch closure and antimalarial drugs [15].

The majority of children with duodenal perforation present with abdominal pain and signs of peritonitis [12]. Although endoscopy can accurately diagnose peptic ulcer disease, the availability in resource-limited hospitals and its application in a pediatric population still remains a challenge in developing countries [14]. Perforation can be diagnosed clinically supported by X-ray abdomen and ultrasound abdomen.

The theory proposed for duodenal perforation in malaria is due to physiological stress which results in secondary peptic ulcer disease and perforation. Malarial parasites mostly infect mature enterocytes of the duodenal villi and the host's inability to eliminate the parasites due to limited systemic and intestinal immunity during illness [10]. Severe dehydration and shock associated with acute diarrhea may cause severe acute bowel ischemia, leading to bowel perforation [12, 13]. It is possible that acute and transient ischemia of the small intestine caused by severe dehydration could have triggered duodenal perforation in our case. In addition, mucosal blood flow has been shown to be lower at anterior duodenal bulb than posterior bulb, suggesting that anterior duodenal bulb may be more susceptible to ischemia, resulting in severe mucosal and mural inflammation and reduced regeneration of the tissues. These factors may contribute to duodenal perforation associated with malaria infection in children.* H. pylori* infection though an important factor leading to perforation in developing countries was not seen in our case. Therefore, greater awareness of the causes of duodenal perforations is of vast importance; clinicians should be aware of these causes to allow for earlier identification of perforation and to facilitate better patient outcomes. The present case report also emphasizes the need for larger studies with severe malaria and duodenal perforation to find the actual cause and effect relationship.

Duodenal perforation is successively treated by repair. Laparotomy has been used extensively for its repair for long time. A minimal invasive laparoscopic surgical repair has been proved to be safe and effective in repair of perforation [16].

## 4. Conclusion

Duodenal perforation in children with malaria is rare, but a severe and potentially lethal complication can occur in those cases. Awareness of the complication in children with malaria is important for appropriate treatment. Early diagnosis and appropriate medical management with antimalarial drugs along with surgical repair can be a lifesaving measure in such cases.
